# Chronic Vomiting and Weight Loss Due to Cecal Pinworm Infestation: A Case Report and Brief Literature Review

**DOI:** 10.7759/cureus.19319

**Published:** 2021-11-06

**Authors:** Hassan Alwafi, Mohammed S Samannodi, Mohammed Almatrafi, Khalil F Miyajan, Ahmed Bishara, Emad M Salawati, Abdullah Y Naser, Mohammed A Almatrafi

**Affiliations:** 1 Pharmacology and Therapeutics, Umm Al-Qura University, Makkah, SAU; 2 Internal Medicine, Umm Al-Qura University, Makkah, SAU; 3 Internal Medicine, King Fahad Armed Forces Hospital, Jeddah, SAU; 4 Medicine and Surgery, Umm Al-Qura University, Makkah, SAU; 5 Medicine, Umm Al-Qura University, Makkah, SAU; 6 Family Medicine Department, Faculty of Medicine, King Abdulaziz University, Jeddah, SAU; 7 Department of Applied Pharmaceutical Sciences and Clinical Pharmacy, Al Isra University, Faculty of Pharmacy, Amman, JOR; 8 Pediatric Infectious Diseases, Umm Al-Qura University, Makkah, SAU

**Keywords:** diarrhea, infection, parasites, pinworms, e. vermicularis

## Abstract

*Enterobius vermicularis* (*E. vermicularis*), also called pinworm or threadworm, is a widespread parasitic infection that has infected approximately 40 million individuals in the United States. However, the infection is rarely seen in the adult population. An atypical presentation of* Enterobius vermicularis* (*E. vermicularis*) infection has been reported previously in children and infants. However, there are no previous reports of this infection among adults in the Middle East. We present a case of a 30-year-old Saudi male patient who presented with significant weight loss, diarrhea, and vomiting. A colonoscopy revealed *E. vermicularis* in the cecum. The patient received one dose of oral albendazole 400 mg and then a repeat dose in two weeks based on his colonoscopy findings. The patient was seen in an outpatient clinic and reported complete resolution of postprandial vomiting. Asking about risk factors if there is any contact with contamination is essential. In addition, colonoscopy and stool analysis may also be considered to confirm the diagnosis. However, future studies investigating the incidence and risk factors of this infection are warranted as similar studies reporting this infection in Saudi Arabia are limited. Proper diagnosis and treatment are also essential to prevent complications of the infection.

## Introduction

*Enterobius vermicularis* (*E. vermicularis*), also called pinworm or threadworm, is a widespread parasitic infection that has infected approximately 40 million individuals in the United States [1].

The infection is more common in children and has affected around 4%-28% of the worldwide child population. However, it is rarely seen in adults [1,2]. Pinworms are approximately 10 mm long and live with their heads attached to the right hemicolon and adjacent to the small intestine. Transmission is usually fecal-oral [3].

*Enterobius vermicularis* infection is usually asymptomatic. The most common presentation is perianal pruritus, watery diarrhea, abdominal pain, and insomnia. However, the infection may progress with complications such as urinary tract infection, weight loss, and infection of the peritoneal cavity, such as appendicitis [4,5]. In addition, *E. vermicularis* is usually associated with eosinophilia and chronic inflammatory infiltrates [6]. *Enterobius vermicularis* is prevalent in young children, mothers with low education levels, and children who do not wash their hands before eating [1].

Previous studies describing this infection are limited with no previous case reports in Saudi Arabia.

## Case presentation

A 30-year-old Saudi male patient presented to our hospital with a six-month history of chronic postprandial vomiting and unintentional weight loss (20 kg). The patient is medically free and is not taking any medications. Moreover, he is a nonsmoker and has denied drinking alcohol or using illicit drugs. He denied sick contact or recent travel.

During his physical examination, his blood pressure was 122/80 mm Hg, his temperature was 37.1°C, his pulse rate was 80 beats per minute, and his respiratory rate was 16 breaths per minute. An abdominal examination revealed mild right lower quadrant tenderness, and the rest of the physical examination was unremarkable.

**Figure 1:**
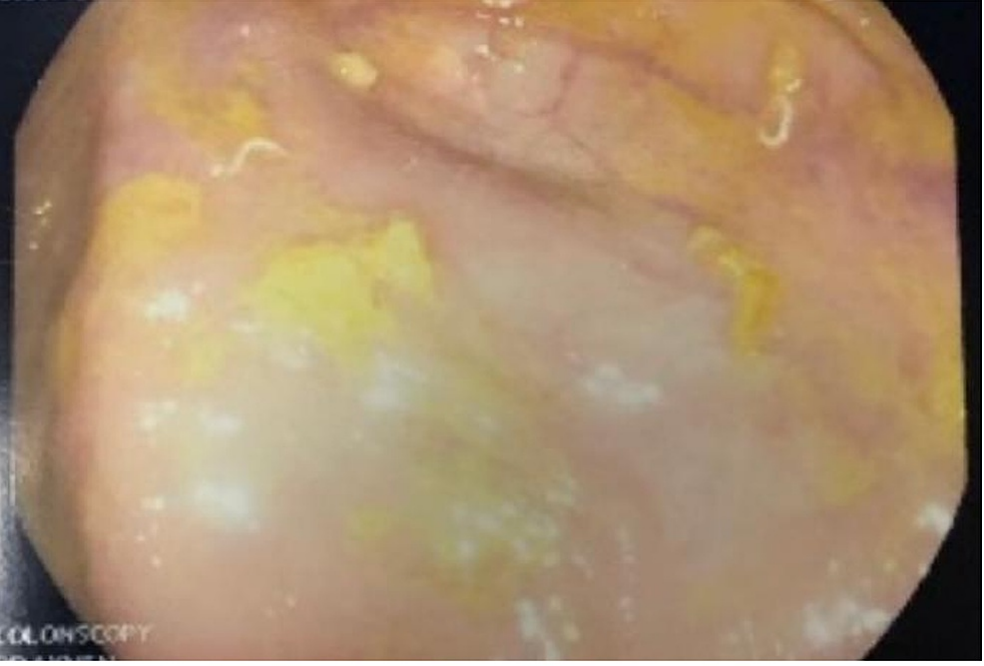
Colonoscopy showing pinworms in the cecum

A complete blood count showed a WBC count of 8500/mm^3^ with 14% eosinophils, hemoglobin of 13.5 g/dL, 39.9% hematocrit, and platelets of 330,000/μL. A complete metabolic panel, including liver and renal functions, revealed nothing noteworthy. During hospitalization, CT abdomen and esophagogastroduodenoscopy were performed with unremarkable results. Due to the right lower quadrant tenderness, a colonoscopy was performed and showed pinworms in the cecum (Figure 1). Stool analysis revealed no evidence of ova or parasites. Based on the colonoscopy findings, we gave the patient one dose of oral albendazole 400 mg and then a repeat dose in two weeks. The patient was seen in an outpatient clinic and reported full resolution of postprandial vomiting.

## Discussion

This case illustrates the successful diagnosis and treatment of an adult patient with pinworms in the cecum, given that we gave the patient one dose of oral albendazole. The patient reported a complete resolution of postprandial vomiting in the outpatient clinic.

In 2019, a case of a 32-year-old female infected with *E. vermicularis* was reported in South Korea. The patient complained of anal pruritus, bleeding, and constipation. Moreover, she had a non-tender abdomen and hemorrhoids [7]. Ours involved a 30-year-old male who presented with a six-month history of chronic postprandial vomiting and mild tenderness in the right lower quadrant.

Appendiceal colic causes right lower quadrant and pelvic pain. According to the appendiceal lumen obstruction hypothesis, when the eggs hatch, the larvae are released into the small intestine [8]. Adult worms are mainly found in the cecum and appendix, where they cause obstruction [8]. This may account for our patient's atypical presentation, chronic postprandial vomiting, and mild right lower quadrant tenderness.

The physical assessment is not specific to distinguish parasitic from nonparasitic appendiceal pain [8]. Before surgery, a thorough history taking and investigation should be performed to look for related symptoms that could indicate pinworm infection, such as anal pruritus and eosinophilia, to improve diagnostic certainty [8]. In cases of suspected appendicitis, ultrasound and CT have proven to be beneficial. Since McBurney can be positive for pinworm when the worms are firmly adherent to the mucosa by their heads, a partial cutting of the appendix with traction will enable inspection of the lumen [9]. If the worms are visible within the luminal cavity, careful thermal dissection with the tip of the scissors directly on the worms or endoscopic suction should be performed [8].

In the study of Kang and Jee, the laboratory results revealed a low level of hemoglobin (11.2 g/dL) and an average white blood cell count of 8600/mm^3^ with 0.9% eosinophils [7]. A colonoscopy revealed a 1-cm worm moving in the anus. However, in our case, the complete blood count showed a WBC count of 8500/mm^3^ with 14% eosinophils, hemoglobin of 13.5 g/dL, 39.9% hematocrit, and platelets of 330,000/μL. The complete metabolic panel, including liver and renal functions, was unremarkable.

During hospitalization, CT abdomen and esophagogastroduodenoscopy were performed with unremarkable results. Due to unexplained right lower quadrant tenderness, a colonoscopy was performed and showed pinworms in the cecum. Stool analysis revealed no evidence of ova or parasites. In the report of Kang and Jee, the patient's five-year-old daughter also reported anal pruritus and had classmates recently diagnosed with pinworm infection [7]. All family members were treated with oral albendazole, and two months later, the anal pruritus disappeared. Based on the colonoscopy findings in our case, the patient received albendazole 400 mg and then a repeat dose in two weeks, and the patient's symptoms were relieved. The CDC [10] recommends mebendazole, pyrantel pamoate, or albendazole to treat pinworm. A single dose is given first, followed by a second dose two weeks later. Pyrantel pamoate is an over-the-counter medication. Medication does not reliably kill pinworm eggs. Consequentially, the second dose prevents reinfection by adult worms hatched from untreated eggs [10].

The life cycle of *E. vermicularis* begins with egg deposition on the perianal folds by gravid adult female worms. Autoinfection occurs when the perianal area is scratched and infective eggs are transferred to the mouth with contaminated hands [8]. This cycle explained the patient's symptoms.

Adults may present with an atypical *E. vermicularis* infection. It is critical to enquire about risk factors in the event of contamination contact. Additionally, colonoscopy and stool analysis could be used to confirm the diagnosis. However, further research addressing the incidence and risk factors for this infection is necessary.

## Conclusions

Finally, we conclude that *E. vermicularis* can be presented in adults with an atypical presentation. Asking about risk factors if there is any contact with contaminants is necessary. Colonoscopy and stool analysis may also be considered to confirm the diagnosis. However, future studies investigating this infection's incidence and risk factors are warranted as similar studies reporting this infection in Saudi Arabia are limited. Proper diagnosis and treatment are also essential to prevent complications of the infection.
